# Vital Sign Prediction of Adverse Maternal Outcomes in Women with Hypovolemic Shock: The Role of Shock Index

**DOI:** 10.1371/journal.pone.0148729

**Published:** 2016-02-22

**Authors:** Alison M. El Ayadi, Hannah L. Nathan, Paul T. Seed, Elizabeth A. Butrick, Natasha L. Hezelgrave, Andrew H. Shennan, Suellen Miller

**Affiliations:** 1 Bixby Center for Global Reproductive Health, Department of Obstetrics, Gynecology and Reproductive Sciences, University of California San Francisco; San Francisco, California, United States of America; 2 Women’s Health Academic Centre, King’s College London,London, United Kingdom; Georgia Regents University, UNITED STATES

## Abstract

**Objective:**

To determine the optimal vital sign predictor of adverse maternal outcomes in women with hypovolemic shock secondary to obstetric hemorrhage and to develop thresholds for referral/intensive monitoring and need for urgent intervention to inform a vital sign alert device for low-resource settings.

**Study Design:**

We conducted secondary analyses of a dataset of pregnant/postpartum women with hypovolemic shock in low-resource settings (n = 958). Using receiver-operating curve analysis, we evaluated the predictive ability of pulse, systolic blood pressure, diastolic blood pressure, shock index, mean arterial pressure, and pulse pressure for three adverse maternal outcomes: (1) death, (2) severe maternal outcome (death *or* severe end organ dysfunction morbidity); and (3) a combined severe maternal and critical interventions outcome comprising death, severe end organ dysfunction morbidity, intensive care admission, blood transfusion ≥ 5 units, or emergency hysterectomy. Two threshold parameters with optimal rule-in and rule-out characteristics were selected based on sensitivities, specificities, and positive and negative predictive values.

**Results:**

Shock index was consistently among the top two predictors across adverse maternal outcomes. Its discriminatory ability was significantly better than pulse and pulse pressure for maternal death (p<0.05 and p<0.01, respectively), diastolic blood pressure and pulse pressure for severe maternal outcome (p<0.01), and systolic and diastolic blood pressure, mean arterial pressure and pulse pressure for severe maternal outcome and critical interventions (p<0.01). A shock index threshold of ≥ 0.9 maintained high sensitivity (100.0) with clinical practicality, ≥ 1.4 balanced specificity (range 70.0–74.8) with negative predictive value (range 93.2–99.2), and ≥ 1.7 further improved specificity (range 80.7–90.8) without compromising negative predictive value (range 88.8–98.5).

**Conclusions:**

For women with hypovolemic shock from obstetric hemorrhage, shock index was consistently a strong predictor of all adverse outcomes. In lower-level facilities in low resource settings, we recommend a shock index threshold of ≥ 0.9 indicating need for referral, ≥ 1.4 indicating urgent need for intervention in tertiary facilities and ≥ 1.7 indicating high chance of adverse outcome. The vital sign alert device incorporated values 0.9 and 1.7; however, all thresholds will be prospectively validated and clinical pathways for action appropriate to setting established prior to clinical implementation.

## Introduction

Approximately 6% of deliveries are complicated by obstetric hemorrhage [1]. Despite a well-established evidence-base for clinical management of obstetric hemorrhage, it remains the leading cause of maternal mortality and morbidity globally [2, 3]. The greatest burden of obstetric hemorrhage is in low-resource settings [2] where deaths occur due to delays in diagnosis and management. Prompt identification and treatment are crucial to reduce hemorrhage-related maternal mortality and morbidity.

Visual estimation of blood loss is routinely used to assess severity and guide resuscitation; however, blood loss is often underestimated [4]. Vital signs monitoring is key to hemodynamic assessment [4], with thresholds for systolic blood pressure (SBP) and pulse used in clinical trigger or early warning systems to prompt intervention [5–8]. Impending shock may be masked by the hemodynamic changes of pregnancy, making conventional vital signs less useful [9], and signs taken in isolation may miss impending deterioration.

Within the general population, shock index (SI), the ratio of pulse to SBP, is proposed as an earlier marker of hemodynamic compromise than conventional vital signs [10]. Trauma literature suggests normal adult SI ranges 0.5–0.7 [11]. A few small studies have evaluated SI within obstetric populations [12–15], none in low or middle income countries, and further research is necessary to inform the clinical utility of SI as an early marker of shock, due to antepartum and peripartum circulatory changes.

We sought to determine the optimal vital sign predictor for severe adverse maternal outcomes among women in hypovolemic shock secondary to obstetric hemorrhage, and to develop thresholds corresponding to early detection triggers for immediate referral or intensive treatment for incorporation into a semi-automated handheld, vital sign alert (VSA) device [16–18] which will be prospectively validated in subsequent work. The device would incorporate a traffic-light early warning system for shock in low-resource settings where delays in detection of impending compromise adversely affect maternal health.

## Materials and Methods

We compiled data from 967 women comprising pre-intervention/control participants from four studies conducted by the Safe Motherhood Program at the University of California, San Francisco that evaluated the effectiveness of the non-pneumatic anti-shock garment (NASG) to reduce adverse maternal outcomes for women with hypovolemic shock secondary to severe obstetric hemorrhage: Egypt 2004 (n = 158), Egypt 2006–2008 (n = 432), Nigeria 2004–2007 (n = 182) and Zambia and Zimbabwe 2007–2012 (n = 195). Detailed descriptions of these studies have been published elsewhere [19–21]. Three of these studies, based at the tertiary level, followed a quasi-experimental design where a pre-intervention period was temporally followed by an NASG intervention period, and one was a cluster-randomized control trial (CRCT) of NASG application at the primary health clinic (PHC) level, prior to transport to tertiary facility for definitive treatment. The pre-intervention/control participants in all studies received standardized evidence-based hemorrhage and shock management [22]. Women in all trials were eligible for study participation if they reached a threshold estimated blood loss and one or more of the following: SBP ≤ 100 mm Hg and/or pulse ≥ 100 BPM. In the tertiary facility studies in Egypt and Nigeria, the threshold estimated blood loss was >750ml, while in the Zambia and Zimbabwe PHC-enrolled study the threshold EBL was >500 mL. The majority of facilities were under-staffed, under-resourced, and characterized by long delays in obtaining definitive care (surgery, blood transfusions). As a secondary analysis of de-identified data, no further institutional review board approval was required for the current analysis. Initial study protocols, including informed consent procedures, were approved by institutional review boards at the University of California, San Francisco, and for each study, respectively, by the following institutions: University of Zambia, Lusaka Research Ethics Committee; Medical Research Council of Zimbabwe; Department of Reproductive Health and Research of the World Health Organization Ethics Review Committee; National Reproductive Health Research Committee of the Nigerian Federal Ministry of Health, El Galaa Maternity Teaching Hospital; Assiut University Women’s Health Center; Alexandria University Teaching Hospital; and Al Minya University Teaching Hospital. All women provided written or thumbprint (if illiterate) informed consent for study participation; all ethics committees provided a waiver of consent from women who were unconscious or confused at time of admission until they recovered or written consent was obtained from a relative as proxy.

We excluded 1 individual who participated in the Zambia trial but was lost to follow-up and had no outcome data for death, and 8 individuals across all studies that had no vital sign data recorded or whose vital signs were indicated as non-palpable. Participants missing other predictors or outcomes were retained, resulting in a final analytic sample of 958 participants.

### Measures

The data analyzed were based on data captured from the parent studies in which, upon enrollment, patients’ vital signs, level of consciousness, urine output, blood loss, IV fluids, blood products transfused and uterotonics administered were recorded at 15 minute intervals, until the cause of bleeding was identified and treated, vital signs were stable (SBP≥100mm Hg, pulse<100 BPM) for at least two hours, and blood loss had decreased to approximately 25–50 mL per hour. Predictor variables for this analysis were values at the measurement interval with the highest SI (pulse/SBP) within the first hour after study entry. Variables included pulse, SBP, diastolic blood pressure (DBP), mean arterial pressure (MAP = (2 x DBP + SBP)/3), SI (pulse/SBP), and pulse pressure (SBP-DBP). Severe shock at study entry was defined as MAP less than 60 mmHg, below which perfusion of vital organs has been proposed to be inadequate [23–25]. BP was measured via an automated blood pressure device or auscultatory technique with mercury sphygmomanometer.

Outcomes comprised any severe adverse maternal event related to obstetric hemorrhage, and included organ system dysfunction-based criteria and intervention-based criteria. Although the outcomes for the original trials were determined prior to the development of the WHO “Near-Miss” criteria [26], they are very similar. The original outcomes for the studies were maternal mortality; end-organ system failure morbidity defined as clinically-diagnosed major organ failure (respiratory, renal, neurological, cardiac) lasting for 24 hours post-resuscitation; and the intervention variables ICU admission, blood transfusion, and emergency hysterectomy for intractable uterine atony. These outcomes were selected because in the majority of our study sites, laboratory-based criteria (e.g., determining DIC by platelets), were not consistently available. For the purposes of the present analysis we used the WHO maternal near-miss indicators for our outcomes [26]. We evaluated maternal status as 1) death or 2) severe maternal outcome (SMO), a composite indicator of death or severe end-organ failure maternal morbidity. Finally we combined SMO with the WHO intervention-based near-miss criteria ICU admission, blood transfusion ≥5 units and emergency hysterectomy (uterine atony diagnoses only). WHO labels these as “critical interventions”; therefore, to be consistent with WHO criteria, we called our composite indicator of SMO and the critical interventions SMO-CI.

### Analyses

We first evaluated the area under the curve (AUC) and associated 95% confidence interval for each of the predictors with each of the maternal outcomes using non-parametric receiver-operating curve (ROC) analysis [27]. We then tested for predictor equality of AUCs across the three outcomes using chi-square tests adjusted by Bonferroni correction for multiple comparisons [28]. To evaluate the impact of having selected one ‘worst’ data point within the first hour, we conducted a sensitivity analysis assessing the AUC of each predictor and outcome combination using the following data points: 1) highest shock index (highest pulse and lowest systolic blood pressure; 2) highest pulse; 3) lowest systolic blood pressure; and 4) lowest mean arterial pressure. We calculated sensitivities, specificities, and positive and negative predictive values for four potential thresholds of SI to select the two optimal thresholds indicating need for referral to higher-level care and need for intensive treatment. The two potential lower thresholds were selected based on proposed thresholds from the literature [11, 13, 15, 29, 30] combined with review of false positives below each threshold from our data. The two upper thresholds were derived from the 95%, 98% and 99% specificity centiles of our outcomes, and a previous analysis of severe postpartum blood loss among women in a higher resourced setting [15]. The thresholds were picked to allow flexibility in clinical utility: the lower threshold to allow a rule out test (i.e., high sensitivity and negative prediction) while the higher threshold an optimal rule in test (i.e. high specificity and positive prediction). Results were not adjusted for the different treatments received by study participants. We conducted sensitivity analyses to determine whether our AUC results were robust across the two contexts in which participants entered our study: at the PHC (Zambia and Zimbabwe) and the tertiary care facility level (Egypt and Nigeria), through modeling interaction terms for vital sign predictors by context within a series of logistic regression models. All analyses were conducted using Stata version 12.0 (StataCorp, College Station, TX). Differences were considered statistically significant where p<0.05.

## Results

Demographics and participants’ status at the time of study entry are presented in Table 1. The mean age of study participants was 28.3 (SD 6.4) and median parity was 2 (IQR 1–4). At study entry, median estimated blood loss was 1000 mL (IQR 750–1500). Most participants entered the study in normal consciousness (57.7%) and 41.6% had altered consciousness. The most common hemorrhage etiologies were uterine atony (32.9%), complications of abortion (15.5%), retained placenta (12.0%) and ectopic pregnancy (10.6%). One-quarter (24.4%) were in severe shock (MAP<60 mmHg).

**Table 1:** Participant Characteristics.

Characteristic	n	%
Age ^c^	28.3 (6.4) ^a^	
Parity ^d^	2 (1–4) ^b^	
Level of Consciousness		
Normal	550	57.7
Altered	397	41.6
Under anesthesia	7	0.7
Estimated Blood Loss at Study Entry ^e^	1000 (750–1500) ^b^	
Hemorrhage Diagnosis		
Uterine Atony	315	32.9
Complications of Abortion	148	15.5
Retained Placenta	115	12.0
Ectopic Pregnancy	104	10.9
Abruptio Placenta	84	8.8
Ruptured Uterus	51	5.3
Placenta Previa	49	5.1
Lacerations	40	4.2
Other	28	2.9
Molar Pregnancy	12	1.3
Missing	6	0.6
Placenta Accreta	6	0.6
MAP<60^c^	233	24.4

MAP: mean arterial pressure.

^a^Mean (SD)

^b^Median (Quartiles)

^c^ n = 956

^d^n = 935

^e^n = 640.

Distributions of vital signs at study entry are presented in Table 2. The proportion of women who developed each outcome was as follows: death (n = 39, 4.1%), SMO (n = 63, 6.6%), SMO-CI (n = 150, 15.7%).

**Table 2:** Distribution of Vital Sign Values.

Vital Sign	N	Median (IQR)
SI	952	1.3 (1.1–1.5)
Pulse	956	117 (110–122)
Systolic BP	954	90 (80–100)
Diastolic BP	863	59 (50–60)
MAP	863	68.7 (60–73.3)
Pulse Pressure	863	34 (30–40)

SI: shock index; BP: blood pressure; MAP: mean arterial pressure.

**Fig 1:**
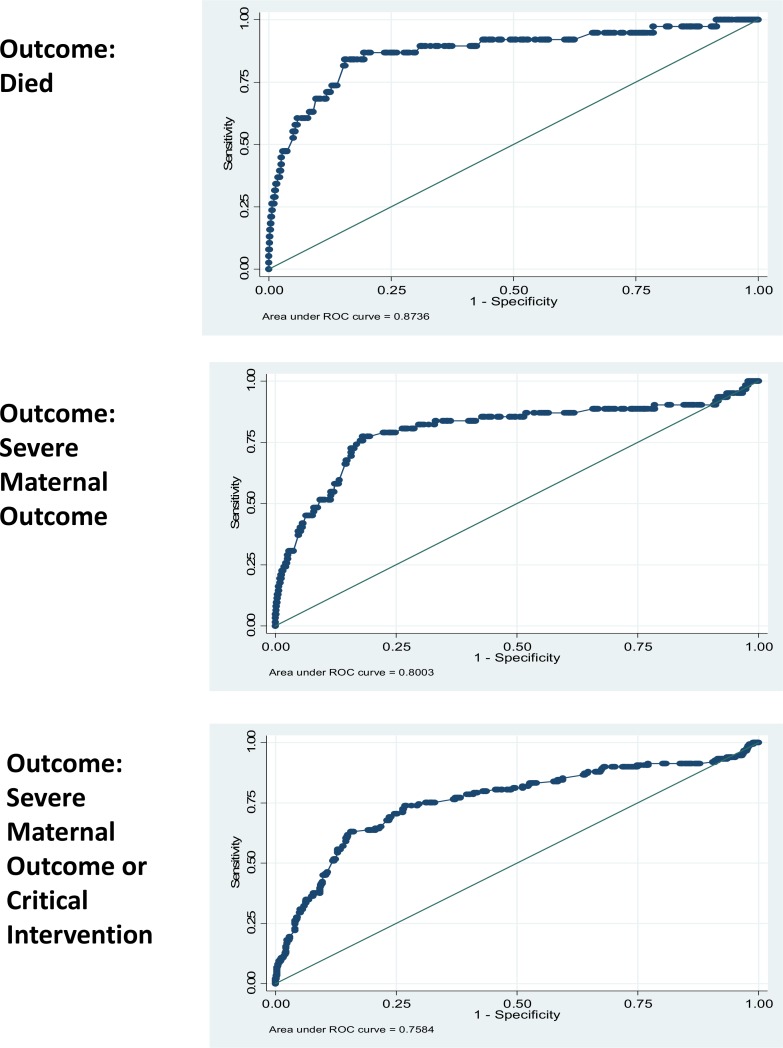
ROC Curves of Shock Index on Outcomes: Died, SMO, and SMO-CI.

Table 3 presents the performance of each vital sign in predicting each of the three adverse outcomes. For death, SI and SBP had the highest AUC value at 0.87 (95% CI 0.80–0.94), which was significantly higher than pulse (p<0.05) and pulse pressure (p<0.01). For SMO, SI and pulse had the highest AUC value at 0.80 (95% CI 0.73–0.87) and 0.80 (95% CI 0.74–0.86), respectively, and were significantly higher than DBP and pulse pressure (p<0.01). Pulse and SI had the highest AUC values for SMO-CI, at 0.80 (95% CI 0.76–0.83) and 0.76 (95% CI: 0.71–0.81), respectively. For SMO-CI, the AUC for SI was significantly higher than for SBP, DBP, MAP and pulse pressure (p<0.01). We chose to develop our vital sign thresholds based on SI because it was one of the top two vital sign predictors across all three maternal status and critical clinical intervention outcomes. Fig 1 presents the ROC curves for shock index across the three outcomes. Our sensitivity analyses indicated that AUC values did not differ significantly across study entry context (not shown). While the AUC results did not support SI as the superior predictor across all adverse maternal outcomes, the combined pattern of magnitude and statistical difference suggests it is the most consistent predictor.

**Table 3:** AUC Values (95% Confidence Intervals) of Vital Sign Discrimination Accuracy for Adverse Maternal Outcomes.

Vital Sign	N	Death	SMO	SMO-CI
**SI**	952	0.87 (0.80–0.94)	0.80 (0.73–0.87)	0.76 (0.71–0.81)
**Systolic BP**	954	0.87 (0.80–0.94)	0.77 (0.70–0.85)	0.69 (0.64–0.75)^↓↓^
**Diastolic BP**	863	0.81 (0.71–0.90)	0.69 (0.63–0.74) ^↓↓^	0.69 (0.63–0.75) ^↓↓^
**Pulse**	956	0.78 (0.69–0.87) ^↓^	0.80 (0.74–0.86)	0.80 (0.76–0.83)
**MAP**	863	0.83 (0.76–0.91)	0.76 (0.68–0.84)	0.70 (0.65–0.76)^↓↓^
**Pulse Pressure**	863	0.39 (0.26–0.51) ^↓↓^	0.51 (0.42–0.60) ^↓↓^	0.50 (0.45–0.56)^↓↓^

AUC: area under the curve; SI: shock index; BP: blood pressure; MAP: mean arterial pressure; SMO: severe maternal outcome (death or severe end-organ failure maternal morbidity); SMO-CI: severe maternal outcome or critical intervention (intensive care unit admission, blood transfusion ≥5 units or emergency hysterectomy).

Results of significance testing for equality of AUCs using Bonferroni-adjusted chi-square test, with SI as reference: ^↓^ Significantly worse than SI (P<0.05)

and ^↓↓^ Significantly worse than SI (P<0.01).

The proportion of women with SI greater than each of the thresholds evaluated is as follows: SI ≥ 0.7: 99.6%, SI ≥ 0.9: 95.0%, SI ≥ 1.4: 32.3% and SI ≥ 1.7: 13.8% (not shown). The performance of SI ≥ 0.7, the upper limit of normal SI for the non-pregnant population [29], and SI ≥ 0.9 (equivalent to pulse 100/110 SBP), the proposed upper limit of normal SI immediately postpartum [13], are presented in Table 4. At SI ≥ 0.7, sensitivity for all adverse outcomes is very high (100.0) and specificity is very low (range 0.4–0.5), indicating that nearly all positives are correctly identified as such, while many negatives are classified as false positives. Sensitivities are slightly lower (range 93.9–100.0) and specificities are slightly higher (range 4.9–5.3) at SI ≥ 0.9 compared to SI ≥ 0.7; very few study participants had SI < 0.7 (0.4%) or SI < 0.9 (5.2%). SI ≥ 0.9 performed better than or similarly to SI≥ 0.7 for all outcomes, thus SI 0.9 was chosen as the lower of the two action thresholds, indicating need for referral to tertiary care or intensive monitoring within tertiary care. As all of the women in the study had hypovolemic shock, the high rate of positive test results is clinically acceptable.

**Table 4:** Performance of SI≥0.7 and SI≥0.9 in Predicting Adverse Maternal Outcomes.

Outcome	SI Threshold	Sensitivity (95% CI)	Specificity (95% CI)	PPV (95% CI)	NPV (95% CI)	Prevalence % (n)
Died	SI≥0.7	100.0 (90.7–100.0)	0.4 (0.1–1.1)	4.0 (2.8–5.5)	100.0 (39.8–100.0)	4.1 (39)
	SI≥0.9	100.0 (90.7–100.0)	5.3 (3.9–6.9)	4.2 (3.0–5.7)	100 (92.6–100.0)	
SMO	SI≥0.7	100.0 (94.2–100.0)	0.5 (0.1–1.2)	6.6 (5.1–8.3)	100.0 (39.8–100.0)	6.6 (63)
	SI≥0.9	95.2 (86.5–99.0)	5.1 (3.7–6.7)	6.5 (5.0–8.4)	93.8 (82.8–98.7)	
SMO-CI	ISI≥0.7	100.0 (97.6–100.0)	0.5 (0.1–1.3)	15.7 (13.5–18.2)	100.0 (39.8–100.0)	15.7 (150)
	SI≥0.9	94.0 (88.8–97.2)	4.9 (3.5–6.6)	15.5 (13.2–18.0)	81.3 (67.4–91.1)	

SI: shock index; SMO: severe maternal outcome (death or severe end-organ failure maternal morbidity); SMO-CI: severe maternal outcome or critical intervention (intensive care unit admission, blood transfusion ≥5 units or emergency hysterectomy).

Table 5 presents the SI centile values at 60%, 80%, 95%, 98% and 99% specificity. Using common values from Table 5, we selected thresholds of SI≥ 1.4 and SI≥ 1.7 for further analysis. Examples of vital sign combinations that represent these thresholds are as follows: pulse 112 and SBP 80 for SI 1.4, and pulse 130 combined with SBP 70 for 1.7. The performance of these thresholds to identify women at highest risk of adverse maternal outcome is shown in Table 6. Comparing SI≥ 1.4 to SI≥ 1.7, specificity is maximized for all outcomes at SI≥ 1.7 (range 70.0–74.8 vs. range 88.5–90.8, respectively) with a corresponding increase in positive prediction (range 10.7–34.2 vs. range 19.8–43.5%, respectively); while sensitivities are lower at SI≥ 1.7 (range 38.3–68.4 vs. 70.5–86.8, respectively) but with a corresponding negative predictive values of 88.8–98.5. Similarly, slightly higher negative predictive values are achieved with SI≥ 1.4 (range 93.2–99.2) due to the higher sensitivities (range 70.5 to 86.8). The selection of which higher threshold to use according to the data for clinical use or for incorporation into the VSA device in development will depend on the clinical setting and existing referral pathways, as both thresholds SI≥ 1.4 and 1.7 may be important for identifying individuals in need of urgent care and resuscitation, dependent upon context.

**Table 5:** SI Values at Various Levels of Specificity by Adverse Maternal Outcome.

Outcome	60% Specificity	80% Specificity	95% Specificity	98% Specificity	99% Specificity
	(95% CI)	(95% CI)	(95% CI)	(95% CI)	(95% CI)
Died	1.38 (1.36–1.40)	1.58 (1.55–1.61)	1.91 (1.86–1.95)	2.10 (2.04–2.16)	2.24 (2.17–2.30)
SMO	1.37 (1.35–1.39)	1.57 (1.54–1.60)	1.89 (1.85–1.94)	2.08 (2.12–2.13)	2.21 (2.15–2.28)
SMO-CI	1.35 (1.33–1.37)	1.53 (1.51–1.56)	1.83 (1.79–1.87)	2.00 (1.95–2.06)	2.13 (2.07–2.19)

SI: shock index; SMO: severe maternal outcome (death or severe end-organ failure maternal morbidity); SMO-CI: severe maternal outcome or critical intervention (intensive care unit admission, blood transfusion ≥5 units or emergency hysterectomy).

**Table 6:** Performance of SI≥1.4 and SI≥1.7 in Predicting Adverse Maternal Outcomes.

Outcome	SI Threshold	Sensitivity (95% CI)	Specificity (95% CI)	PPV (95% CI)	NPV (95% CI)	Prevalence % (n)
Died	SI≥1.4	86.8 (71.9–95.6)	70.0 (66.9–73.0)	10.7 (7.5–14.8)	99.2 (98.2–99.7)	4.1 (39)
	SI≥1.7	68.4 (51.3–82.5)	88.5 (86.3–90.5)	19.8 (13.4–27.7)	98.5 (97.5–99.2)	
SMO	SI≥1.4	80.6 (68.6–89.6)	71.4 (68.3–74.3)	16.4 (12.5–21.1)	98.1 (96.8–99.0)	6.6 (63)
	SI≥1.7	51.6 (38.6–64.5)	88.8 (86.6–90.8)	24.4 (17.3–32.7)	96.3 (94.8–97.5)	
SMO-CI	SI≥1.4	70.5 (62.5–77.7)	74.8 (71.7–77.8)	34.2 (28.9–39.8)	93.2 (91.0–95.0)	15.7 (150)
	SI≥1.7	38.3 (30.4–46.6)	90.8 (88.6–92.7)	43.5 (34.9–52.4)	88.8 (86.4–90.9)	

SI: shock index; SMO: severe maternal outcome (death or severe end-organ failure maternal morbidity); SMO-CI: severe maternal outcome or critical intervention (intensive care unit admission, blood transfusion ≥5 units or emergency hysterectomy)

## Discussion

Our analysis found SI to be the most consistent predictor across a selection of severe maternal outcomes and critical clinical indicators in women with hypovolemic shock secondary to obstetric hemorrhage. High sensitivity combined with clinically practical specificity drove our choice of SI ≥ 0.9 as the threshold for referral to a tertiary facility capable of comprehensive emergency obstetric care or rigorous monitoring within such a context. We chose a higher threshold of SI ≥ 1.4 to indicate need for intensive intervention because this value maximized identification of women needing rapid resuscitation balanced with high sensitivity and negative predictive value. However, for the purposes of clinical validation of the VSA device, an urgent resuscitation threshold of SI ≥ 1.7 was implemented in order reduce the rate of false positives and loss of trust in the VSA device at its inception; however all 3 thresholds will be evaluated prospectively. Such a validation process will help to further refine the higher action threshold, as it may vary based on clinical context and population.

Previous research has suggested a normal SI range of 0.7–0.9 for obstetric populations, with 0.9 representing the transition into abnormality [13]. Research with non-pregnant populations confirms that an SI threshold of 0.9 indicates need for intensive management and a higher risk of mortality [31, 32]. Ectopic pregnancy studies report a high correlation of rupture with similar SI values [12, 33]. One PPH study supported 0.9 as a referral threshold using multiple adverse outcomes [15], and another reported a higher value of 1.1 as indicative of transfusion requirement but suggested 1.0 for simplification of use within an acute obstetric emergency [13]. Within our sample, a threshold of 0.9 had high sensitivity but low specificity; most women with adverse outcomes were identified using this threshold, suggesting that it represents a relevant threshold for medical intervention and referral. The low specificity observed for this threshold is clinically acceptable given our population of women already in hypovolemic shock. The broad range of obstetric hemorrhage etiologies across our participants supports the generalizability of this alert threshold.

The literature on SI threshold indicating highest risk of adverse outcome is sparse. Trauma-related mortality has been found to be significantly increased above SI 1.0, 1.4 and 1.8 [34]. For postpartum hemorrhage, one study previously conducted by our research group within a higher-resource setting selected SI≥1.7 as trigger for intensive resuscitation [15]. This threshold held higher positive predictive value within the current analysis as well, although the outcomes were not as severe in the former study. However, within the current population already meeting the criteria for hypovolemic shock secondary to obstetric hemorrhage, our analysis suggested a slightly lower value, SI≥1.4, to indicate urgent need for resuscitation. Given the long delays that such women in low-resource areas face in transport and receipt of definitive treatment upon arrival at tertiary facilities, this lower threshold prioritizes earlier recognition and more rapid intensive treatment, which is more suitable for such a context. However, as indicated above, both higher thresholds will be prospectively evaluated within a larger facility-based sample.

This study represents a preliminary evaluation of SI as a predictor of adverse maternal outcomes in women with obstetric hemorrhage/hypovolemic shock in low-resource settings. We draw upon a large sample of women with a variety of obstetric hemorrhage etiologies, and prioritize the most robust and salient outcome of mortality. However, the generalizability of our findings is limited to similar clinical and population contexts, and certain characteristics of our data and analysis represent limitations to our study, which may affect the interpretation of our results. First, all women in the original studies were experiencing hypovolemic shock secondary to severe obstetric hemorrhage; therefore, they already met the WHO definition of “near-miss” [26]. Second, improved prediction capability is typically achieved with higher prevalence rates than our most robust outcomes, death and SMO [35]. Our SMO-CI outcome which includes ICU admission and blood transfusion, is more prevalent, but may be susceptible in low resource settings to supply variability (availability of blood or presence of functioning ICU, or capable staff for ICU), the financial ability of patients to afford interventions, and other human resource constraints prevalent in LMICs [19]. Third, no treatment data were included in this analysis. Some women received IV fluids, uterotonics, procedures and surgical interventions that may have influenced clinical trajectories between study entry and outcome. Future studies on prediction of adverse outcome should include timing and impact of therapies on resuscitation. Fourth, our vital sign measures were taken at the combined point of highest pulse and lowest systolic blood pressure (i.e., highest shock index) within the first hour after study entry which may have inadvertently biased our selection of shock index as the most discriminatory predictor of adverse outcome. We evaluated this potential for bias within a sensitivity analysis of AUC across four “worst” categories: shock index, pulse, systolic blood pressure and MAP and report no substantial or consistent patterning; however, this remains a potential limitation. Furthermore, our vital sign measures represent only one time point; subsequent work on shock index should utilize repeated measures for a more robust analysis. Fifth, we included all individuals with data for each comparison, instead of conducting a complete case analysis; thus, comparisons across each of the predictor and outcome combinations are affected by slightly different missing data patterns. Sixth, we excluded several participants who were in the most severe condition due to non-palpable vital signs, thus our analysis is more conservative. Finally, the impetus for this analysis was to inform thresholds for a hand-held semi-automated early warning VSA device incorporating a traffic-light warning system for hypertension and shock designed for low-resource settings [16, 17]. Such a device can be utilized to improve referral practices even among low-level community health providers including traditional birth attendants; therefore, we did not consider ease of calculation in our threshold development [13, 36]. As suggested by Le Bas et al [13], recommending that referral or intervention be triggered where pulse is greater than or equal to systolic blood pressure, indicating an SI threshold of 1.0, may be useful in settings where health care workers are unable to compute the ratio of pulse to SBP.

The developing evidence base on the utility of SI, strengthened by the results from our analysis, indicates a potential role for SI in early diagnosis and management of shock and for reducing adverse outcomes in obstetric populations. We suggest a lower SI threshold of 0.9 indicating need for referral to tertiary facility or rigorous monitoring within tertiary care. Based on our results, we suggest higher SI thresholds of 1.4 to indicate the urgent need for intensive treatment, and 1.7 as indicative of high risk of adverse event. Further research should evaluate these thresholds prospectively and focus on implementing SI as a tool at multiple clinical levels with different categories of care providers to maximize its utility within clinical obstetric early warning systems [17, 18].

## References

[1] Carroli G, Cuesta C, Abalos E, Gulmezoglu AM. Epidemiology of postpartum haemorrhage: a systematic review. Best Practice & Research Clinical Obstetrics & Gynaecology. 2008;22(6):999–1012. 18819848 10.1016/j.bpobgyn.2008.08.004

[2] Say L, Chou D, Gemmill A, Tunçalp Ö, Moller A-B, Daniels J, et al Global causes of maternal death: a WHO systematic analysis. The Lancet Global Health. 2014;2(6):e323–e33. 10.1016/S2214-109X(14)70227-X 25103301

[3] AbouZahr C. Global burden of maternal death and disability. Br Med Bull. 2003;67:1–11. Epub 2004/01/09. 14711750 10.1093/bmb/ldg015

[4] Schorn MN. Measurement of blood loss: review of the literature. J Midwifery Womens Health. 2010;55(1):20–7. Epub 2010/02/05. 10.1016/j.jmwh.2009.02.014 20129226

[5] Kim Gregory, Elliott Main, Audrey Lyndon. Definition, Early Recognition and Rapid Response Using Triggers. 2009.

[6] Al-Foudri H, Kevelighan E, Catling S. CEMACH 2003–5 Saving Mothers' Lives: lessons for anaesthetists. Continuing Education in Anaesthesia, Critical Care & Pain. 2010;10(3):81–7.

[7] Cantwell R, Clutton-Brock T, Cooper G, Dawson A, Drife J, Garrod D, et al Saving Mothers' Lives: Reviewing maternal deaths to make motherhood safer: 2006–2008. The Eighth Report of the Confidential Enquiries into Maternal Deaths in the United Kingdom. BJOG. 2011;118 Suppl 1:1–203. Epub 2011/03/05. 10.1111/j.1471-0528.2010.02847.x 21356004

[8] American College of Obstetricians & Gynecologists. Committee opinion no. 590: preparing for clinical emergencies in obstetrics and gynecology. Obstet Gynecol. 2014;123(3):722–5. Epub 2014/02/21. 10.1097/01.AOG.0000444442.04111.c6 24553170

[9] Bonanno FG. Hemorrhagic shock: The "physiology approach". J Emerg Trauma Shock. 2012;5(4):285–95. Epub 2012/12/19. 10.4103/0974-2700.102357 23248495 PMC3519039

[10] Birkhahn RH, Gaeta TJ, Terry D, Bove JJ, Tloczkowski J. Shock index in diagnosing early acute hypovolemia. Am J Emerg Med. 2005;23(3):323–6. 15915406 10.1016/j.ajem.2005.02.029

[11] Rady MY, Rivers EP, Martin GB, Smithline H, Appelton T, Nowak RM. Continuous central venous oximetry and shock index in the emergency department: use in the evaluation of clinical shock. Am J Emerg Med. 1992;10(6):538–41. Epub 1992/11/01. 1388378 10.1016/0735-6757(92)90178-z

[12] Birkhahn RH, Gaeta TJ, Van Deusen SK, Tloczkowski J. The ability of traditional vital signs and shock index to identify ruptured ectopic pregnancy. Am J Obstet Gynecol. 2003;189(5):1293–6. 14634556 10.1067/s0002-9378(03)00663-x

[13] Le Bas A, Chandraharan E, Addei A, Arulkumaran S. Use of the "obstetric shock index" as an adjunct in identifying significant blood loss in patients with massive postpartum hemorrhage. Int J Gynaecol Obstet. 2014;124(3):253–5. Epub 2014/01/01. 10.1016/j.ijgo.2013.08.020 24373705

[14] Pacagnella RC, Souza JP, Durocher J, Perel P, Blum J, Winikoff B, et al A systematic review of the relationship between blood loss and clinical signs. PLOS One. 2013;8(3):e57594 Epub 2013/03/14. 10.1371/journal.pone.0057594 23483915 PMC3590203

[15] Nathan HL, El Ayadi A, Hezelgrave NL, Seed P, Butrick E, Miller S, et al Shock index: an effective predictor of outcome in postpartum haemorrhage? BJOG. 2015;122(2):268–75. Epub 2014/12/30. 10.1111/1471-0528.13206 25546050

[16] Nathan HL, De Greef A, Hezelgrave NL, Chappell LC, Shennan AH. Accuracy validation of the Microlife 3as1-2 blood pressure device in an African pregnant population with low blood pressure. Blood pressure monitoring. In Press. 10.1097/MBP.0000000000000134 26020367

[17] Nathan HL, de Greeff A, Hezelgrave NL, Chappell LC, Shennan AH. An accurate semiautomated oscillometric blood pressure device for use in pregnancy (including pre-eclampsia) in a low-income and middle-income country population: the Microlife 3AS1-2. Blood pressure monitoring. 2015;20(1):52–5. Epub 2014/09/23. 10.1097/MBP.0000000000000086 25243711

[18] Nathan HL, de Greeff A, Hezelgrave NL, Duhig KE, Chappell LC, Shennan AH. [208-POS]: An accurate semi-automated oscillometric blood pressure device for use in pregnancy, including pre-eclampsia, in a low- and middle-income country population: The Microlife 3AS1-2. Pregnancy hypertension. 2015;5(1):104–5. Epub 2015/03/20. 10.1097/MBP.0000000000000086 25243711

[19] Miller S, Bergel EF, El Ayadi AM, Gibbons L, Butrick EA, Magwali T, et al Non-pneumatic anti-shock garment (NASG), a first-aid device to decrease maternal mortality from obstetric hemorrhage: a cluster randomized trial. PLOS One. 2013;8(10):e76477 Epub 2013/11/07. 10.1371/journal.pone.0076477 24194839 PMC3806786

[20] Miller S, Hamza S, Bray EH, Lester F, Nada K, Gibson R, et al First aid for obstetric haemorrhage: the pilot study of the non-pneumatic anti-shock garment in Egypt. Bjog. 2006;113(4):424–9. Epub 2006/03/24. 16553654 10.1111/j.1471-0528.2006.00873.x

[21] Miller S, Fathalla MM, Ojengbede OA, Camlin C, Mourad-Youssif M, Morhason-Bello IO, et al Obstetric hemorrhage and shock management: using the low technology Non-pneumatic Anti-Shock Garment in Nigerian and Egyptian tertiary care facilities. BMC Pregnancy Childbirth. 2010;10:64 Epub 2010/10/20. 10.1186/1471-2393-10-64 20955600 PMC2966449

[22] WHO. Managing Complications in Pregnancy and Childbirth: A guide for midwives and doctors Geneva, Switzerland: WHO, UNICEF, UNFPA, World Bank; 2003.

[23] McAuley DF. The Clinician’s Ultimate Reference—Mean Arterial Pressure GlobalRPh Inc.; 2005; Available: http://www.globalrph.com/map.htm. Accessed 28 October 2008.

[24] Cecconi M, De Backer D, Antonelli M, Beale R, Bakker J, Hofer C, et al Consensus on circulatory shock and hemodynamic monitoring. Task force of the European Society of Intensive Care Medicine. Intensive care medicine. 2014;40(12):1795–815. Epub 2014/11/14. 10.1007/s00134-014-3525-z 25392034 PMC4239778

[25] Varpula M, Tallgren M, Saukkonen K, Voipio-Pulkki LM, Pettila V. Hemodynamic variables related to outcome in septic shock. Intensive care medicine. 2005;31(8):1066–71. Epub 2005/06/24. 15973520 10.1007/s00134-005-2688-z

[26] World Health Organization. Evaluating the quality of care for severe pregnancy complications: the WHO near-miss approach for maternal health Geneva, Switzerland: World Health Organization, 2011.

[27] Pepe MS. The Statistical Evaluation of Medical Tests for Classification and Prediction Great Britain: Oxford University Press; 2004.

[28] Abdi H. The Bonferroni and Sidak corrections for multiple comparisons In: Salkind N, editor. Encyclopedia of Measurement and Statistics. Thousand Oaks, CA: Sage; 2010.

[29] Rady MY, Nightingale P, Little RA, Edwards JD. Shock index: a re-evaluation in acute circulatory failure. Resuscitation. 1992;23(3):227–34. 1321482 10.1016/0300-9572(92)90006-x

[30] Sohn CH, Kim WY, Kim SR, Seo DW, Ryoo SM, Lee YS, et al An increase in initial shock index is associated with the requirement for massive transfusion in emergency department patients with primary postpartum hemorrhage. Shock. 2013;40(23707978):101–5. 23707978 10.1097/SHK.0b013e31829b1778

[31] Rady MY, Smithline HA, Blake H, Nowak R, Rivers E. A comparison of the shock index and conventional vital signs to identify acute, critical illness in the emergency department. Ann Emerg Med. 1994;24(4):685–90. 8092595 10.1016/s0196-0644(94)70279-9

[32] McNab A, Burns B, Bhullar I, Chesire D, Kerwin A. A prehospital shock index for trauma correlates with measures of hospital resource use and mortality. Surgery. 2012;152(3):473–6. Epub 2012/09/04. 10.1016/j.surg.2012.07.010 22938906

[33] Onah HE, Oguanuo TC, Mgbor SO. An evaluation of the shock index in predicting ruptured ectopic pregnancy. J Obstet Gynaecol. 2006;26(5):445–7. Epub 2006/07/19. 16846874 10.1080/01443610600747314

[34] Sloan EP, Koenigsberg M, Clark JM, Weir WB, Philbin N. Shock Index and Prediction of Traumatic Hemorrhagic Shock 28-Day Mortality: Data from the DCLHb Resuscitation Clinical Trials. The western journal of emergency medicine. 2014;15(7):795–802. Epub 2014/12/11. 10.5811/westjem.2014.7.21304 25493120 PMC4251221

[35] Linden A. Measuring diagnostic and predictive accuracy in disease management: an introduction to receiver operating characteristic (ROC) analysis. Journal of evaluation in clinical practice. 2006;12(2):132–9. Epub 2006/04/04. 16579821 10.1111/j.1365-2753.2005.00598.x

[36] de Greeff A, Nathan H, Stafford N, Liu B, Shennan AH. Development of an accurate oscillometric blood pressure device for low resource settings. Blood pressure monitoring. 2008;13(6):342–8. Epub 2008/11/21. 10.1097/MBP.0b013e32830fd07c 19020425

